# Human cercarial dermatitis (HCD) in the UK: an overlooked and under-reported nuisance?

**DOI:** 10.1186/s13071-024-06176-x

**Published:** 2024-02-22

**Authors:** Orla Kerr, Alexandra Juhász, Sam Jones, J. Russell Stothard

**Affiliations:** 1 Department of Tropical Disease Biology, Liverpool School of Tropical Medicine, Liverpool, L3 5QA UK; 2 Institute of Medical Microbiology, Semmelweis University, Budapest, 1089 Hungary

**Keywords:** Avian schistosomiasis, *Bilharziella*, Outdoor swimming, Public health, *Trichobilharzia*

## Abstract

**Background:**

Human cercarial dermatitis (HCD) is a clinical disease typically caused by skin-penetrative larvae of avian schistosomes. Its geographical epidemiology is firmly tied with that of infected freshwater intermediate snail hosts. To better understand the current distribution of HCD and its level of nuisance in the UK, we undertook a systematic literature review.

**Methods:**

Following PRIMSA guidelines, PubMed and Scopus databases were searched with keywords “human cercarial dermatitis” OR “swimmer’s itch” AND “United Kingdom”. Articles about imported cases of HCD, or HCD outside the UK, were not formally included.

**Results:**

A total of 30 articles were initially identified. A further two were gained by inspection of all citations. After screening, eight publications were analysed where the location, number of cases and putative avian schistosome species incriminated were tabulated. HCD is mainly found in the south of England, though gaps in evidence and reporting remain across the UK.

**Conclusions:**

Despite its noted recent rise in open water swimmers, published literature on HCD across the UK is sparse; this condition is both overlooked and under-reported. We therefore recommend establishing a national database that raises awareness and encourages self-reporting of this nuisance disease.

**Graphical Abstract:**

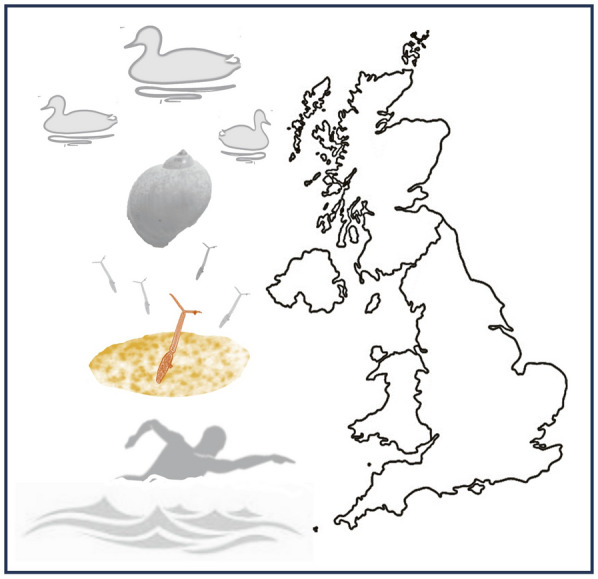

## Background

Nearly one hundred years ago, avian schistosome cercariae were first discovered as the causative agent of cercarial dermatitis in the USA [1]. The life cycle of many avian schistosome species is complex [2, 3]. Of particular note is that avian cercariae are attracted to human skin, penetrating the upper dermal layers in response to key fatty acids also found in duck feet skin [4]. Since humans are accidental ‘dead-end’ hosts, these invading avian cercariae die but in so doing stimulate a strong allergic host reaction; this is pathognomonic for human cercarial dermatitis (HCD), for in the 3–48 h period after exposure, a dermal rash will appear with intensely pruritic papules and erythaema. This is followed by intense itching [4], which gives HCD its more common name of ‘swimmer’s itch’. Although the symptoms of HCD are known, data on associated immune responses in human patients are sporadic and incomprehensive; Macháček et al. [5] attempted to correlate symptoms, personal history and time course of HCD with differential cell counts, dynamics of selected cytokines and dynamics and quality of antibody response [5].

Avian schistosomes are distributed worldwide, save on Antarctica [6]. In Europe and North America, HCD is considered an emerging and/or re-emerging disease as cases are increasing [7]. Indeed, monitoring of HCD outbreaks in Europe is now the subject of numerous local and international research projects; various researchers have attempted to better understand risk by examination of snails, inspection of birds and cercariometry of water [8–13], alongside introduction of environmental DNA surveillance [14]. Whilst the prevalence of schistosomiasis in European birds can reach 38% [6, 8], in the UK its present epidemiology is poorly understood [6, 7] despite the presence of numerous freshwater bodies that harbour infected snails along major bird migration flyways from Europe [15, 16].

In Scotland, for example, Pennycott et al. [17] found mute swans infected with avian schistosomes that might point towards HCD’s most northerly range [17]. Elsewhere in the UK, notable outbreaks of HCD have occurred, where its detrimental impacts on local economies dependent on tourism can take place [18].

Given shifting snail and bird distribution patterns, alongside climate change, as a foundation step towards better surveillance of HCD in the UK, we conducted a systematic review of the formal literature and later investigated contemporary reports of HCD in the national media.

## Methods

### Research selection and search criteria

To collate research articles reporting HCD in the UK, literature searches were conducted in two electronic databases (PubMed and Scopus) on 13 March 2023. Manuscripts were selected based on the following inclusion criteria of studies conducted in UK, reports of HCD cases and avian schistosome species implicated.

**Fig. 1:**
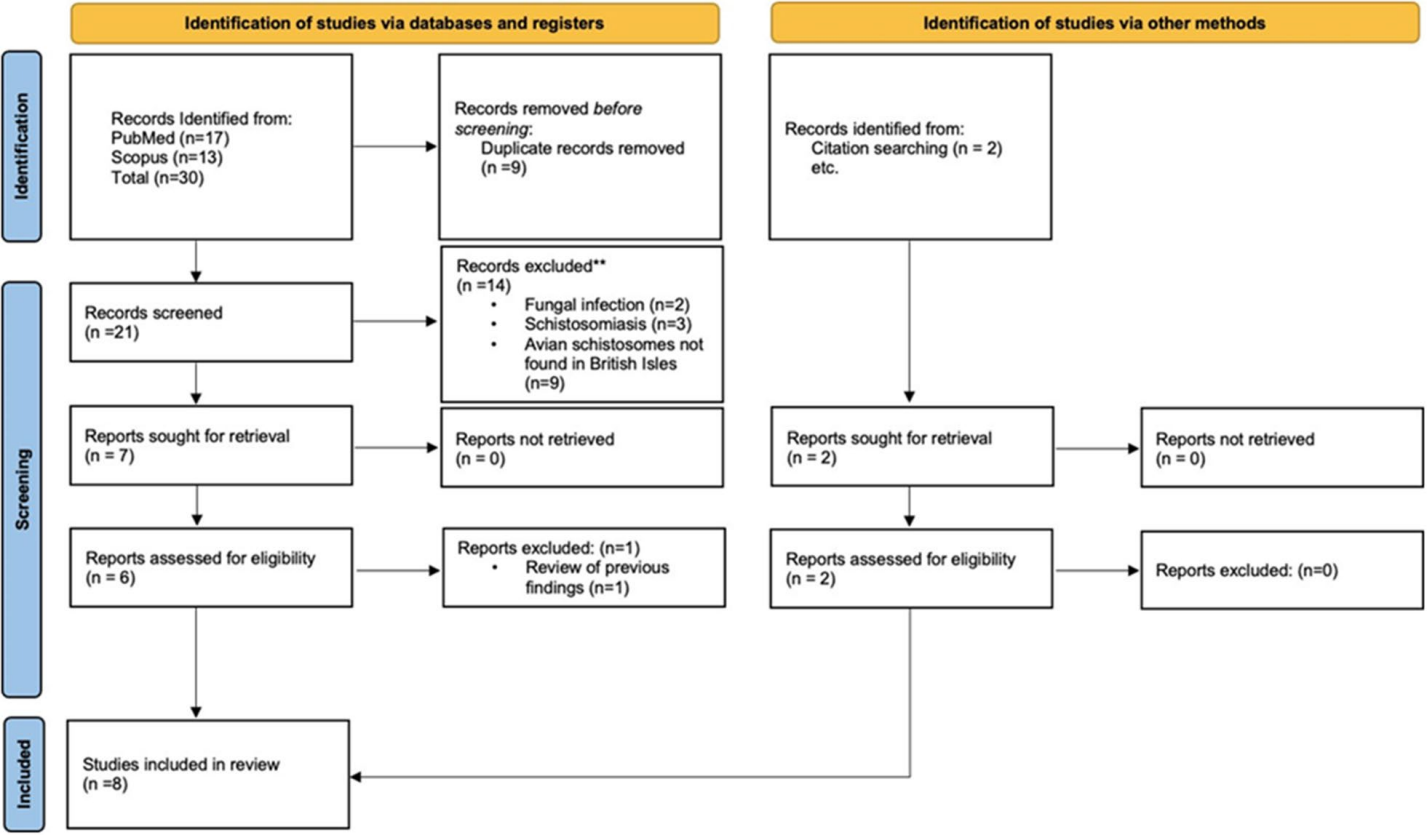
Flow chart of the study selection and identifcation process on PubMed and Scopus. The steps were adopted from the Preferred Reporting Items for Systematic Reviews and Meta-Analyses (PRISMA) guidelines

The detailed search strategies and PRISMA 2020 flow chart are shown in Fig. 1. We focused on keyword of “human cercarial dermatitis” OR "swimmer’s itch” AND “United Kingdom”. The initial results were imported into Mendeley and duplicates removed. The predetermined eligibility criteria were used to exclude any irrelevant articles i.e. imported cases, HCD outside UK and cercariae outside of UK.

### Data extraction

Studies that met our inclusion criteria were subjected to the next phase of screening to extract the following information: month/year of findings; location, main findings such as prevalence if reported, avian schistosome species implicated and year of publication. No time restrictions were placed on publications identified from the systematic literature review.

## Results

In total, 30 studies were retrieved from the customized searches. The systematic review identifed eight articles on HCD in UK that met the inclusion and eligibility criteria. While carrying out the full text screen of these eight articles, two further articles not found through the initial search were identified upon inspection of articles’ citations. However, one article was inaccessible with a message being to the book’s author for retrieval of the relevant extract but with no response. One widely known outbreak of HCD in Norfolk, 2004, was mentioned in several papers but no specific evidence could be found through databases.

All published reports describing cercariae and HCD outbreaks in the UK occurred in the summer months, being always connected with paddling or swimming in infested water. There are several species of cercariae which have been detailed in the articles on HCD in the UK. The majority report on *Trichobilharzia* spp. but with varying species-specific reliability due to a lack of confirmatory molecular analysis. Table 1 shows the key information taken from the selected literature. There appeared to be only a modest number of papers on HCD but these publications frequently noted that as HCD research is increasing there is also a growing number of publications but these were not specifically tied with discrete HCD outbreaks.

**Table 1:** Key information is taken from the selected articles

Localities in UK	Date of findings	Findings	Cercariae species (intermediate host)	References
Roath Park Lake, Cardiff	Summers (1928–1930)	Several hundred people developed HCD	*Trichobilharzia* sp. (*Lymnaea stagnalis*)	[23]
Rickmansworth Aquadrome, Berkshire, England	Summer (1970)	250 people affected by HCD	Cercariae of* “ocellata”* group (*Planorbis aronicus*)	[24]
Southeast England	After summer (1987)	Undisclosed number of reports of HCD after swimming	*None identified*	[32]
Freshwater lake, Ipswich	Summer (1987)	Undefined outbreak of HCD	*None identified*	[33]
Central Scotland	(1996)	Mute swans found infected with avian schistosomes	*None identified*	[17]
Lochore Meadows Country Park, Fife, Scotland	July (2006)	10 children and 2 adults developed HCD	*Trichobilharzia* spp. (*Lymnaea stagnalis*)	[20]
Hampshire, England	August (2011)	Snails found releasing cercariae implicated in HCD	*Trichobilharzia franki* (*Radix auricularia*)	[7]
Knowsley Safari, Prescot, England	July (2021)	Snails found releasing cercariae implicated in HCD	*Bilharziella polonica* (*Planorbarius corneus*) *Trichobilharzia anseri* (*Ampullaceana balthica*)	[19]

## Discussion

**Fig. 2:**
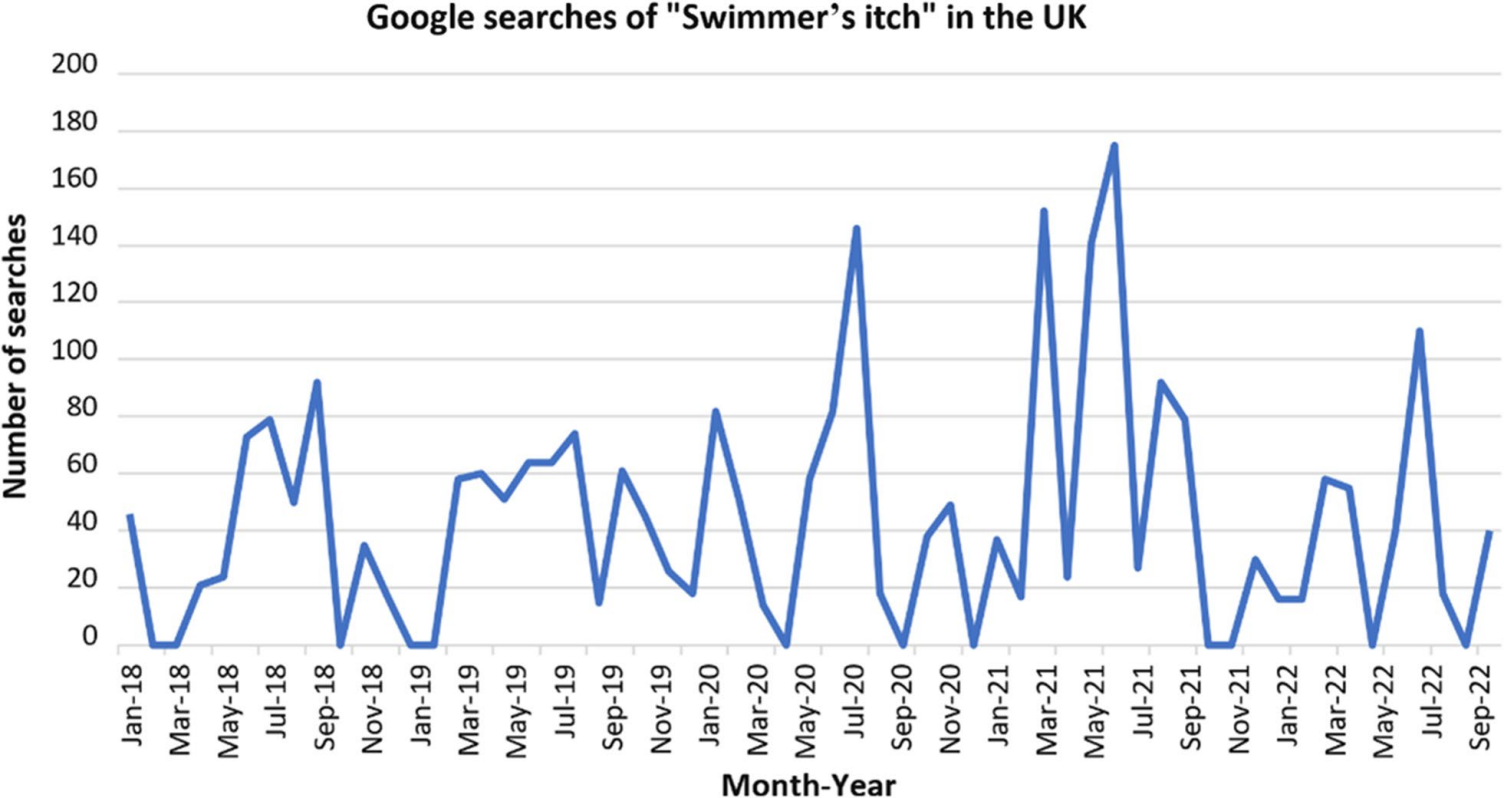
The number of Google searches for “swimmer’s itch” conducted in the UK over the last 5 years. Graph showing the number of the search term “swimmer’s itch” on Google in the UK. There has been increasing popularity in the term over the past 5 years and there are annual peaks in summer months. The term was most popular in July 2021

As shown in Table 1, published literature on HCD in the UK is surprisingly sparse; only eight articles were identified, yet these provide solid epidemiologcial evidence, alongside its underlying putative aetiological agents. All eight reports describing cercariae and HCD outbreaks were observed during summer months only, conclusive of HCD’s well-known seasonality elsewhere [7]. The summer season also influences human behaviour which leads to exposure, outdoor activities such as open water swimming being more common. This clear seasonality can be confirmed with online searches for “swimmer’s itch” in Google, where there are cyclical peaks during summer months each year although such online searches are likely to be by those who wish to know more about what swimmer’s itch actually is (Fig. 2) or whether other animals such as companion pets, for example dogs, are also at risk.

In terms of freshwater snail species implicated, cercarial shedding from *Lymnaea stagnalis* was noted. This was positively correlated with temperature and light with these cercariae exhibiting positive phototaxis [12], most often during the afternoon and/or early evening [19]. It is reasonable to speculate that HCD may become more common in the UK because of increasing rise of water temperatures in recent years. For example, Fraser et al. [20] noted an increased incidence of HCD as described occurred during a period of unusually hot weather [20]. Larsen et al. [21] have suggested that a 0.8 ℃ increase in temperature within the last century may be associated with increased prevalence of *Trichobilharzia* spp. in more northerly parts of Europe [21].

A study conducted in Oxfordshire has also shown that climate change was affecting the migratory behaviour of birds [22]. It is therefore sensible to understand the role of waterfowl on the epidemiology of HCD and to apply appropriate tools to better identify avian schistosome species to assess their risk(s). This is particularly pertinent in view of the distinct lack of knowledge of the diversity of avian flukes and their intermediate freshwater snail hosts in the UK. Fraser et al. [20] identified cercariae morphologically as *Trichobilharzia* species using a “dichotomous key” but did not go on to make any further species-specific ascertions [20].

Harding [23] identified *Trichobilharzia ocellata* through consultation with helminthologists at the British Museum [23], while Knight and Worms (1972) reported cercariae of the “*Ocellata*” group [24]. These descriptions may now be incorrect as the identity of *T. ocellata* has now been called into question. Rudolfová et al. [25] found that various isolates of *T. ocellata* from across the globe were genetically dissimilar and that European isolates of this description are actually identical to *Trichobilharzia szidati* when DNA sequence analysis was conducted [25].

Today, molecular DNA identification is the only reliable method to identify the avian cercariae incriminated in HCD as their morphological features are notiously uniform. Upon application of DNA screening, the phylogenic analysis of cercariae by Lawton et al. [7] implicated *Trichobilharzia franki*. They also noted two novel British lineages of this species which are most closely related to French isolates of *T. franki*. More recently, and using molecular methods, Juhász et al. [13] uncovered greater and surprising diversity within avian schistosomes in the northwest of England. For example, *Bilharziella polonica* was noted, a first report in recent years, alongside *Trichobilharzia anseri* as well as an as of yet divergent lineage within *Trichobilharzia* [19]. Outside of the UK, *Bilharziella* is less commonly associated with HCD but is an avian parasite of some general concern [3].

A key gap arising from our systematic literature search is that no official reporting mechanisms in the UK, for example by the UK Health Security Agency (UKHSA), exist. Thus, the true number of people who seek medical attention for HCD is unknown, a worrysome deficit in satisfactory surveillance, particularly as HCD can be confused with other dermatitis for which central guidance from the UK-NHS exists [20]. Nonetheless, national media have reported on worrisome HCD outbreaks, most recently in 2023 in Llanishen Reservoir, Wales (see https://www.bbc.com/news/uk-wales-66328852.amp) and in 2021 in Loch Lomand, Scotland (see https://www.heraldscotland.com/news/homenews/194102211.loch-lomond-wild-swimmers-warned-parasite-skin-reaction/). Each report led to local restrictions in recreational activities and closure of public access. Developing a reporting method where a physician or member of the public may self-report where they have experienced HCD could be valuable. A reporting system, for example, could be styled like that for Lyme disease [26], where people are encouraged to be tick aware. A similar inititiave for HCD, as advertised on wild swimming websites, where water quality and/or sewage contamination is also noted, could help identify at-risk locations. Here, malacological sampling or perhaps environmental DNA inspections for avian schistosomes [27] could be sensibly applied.

Across the UK, certain councils, for example Oxford, already offer HCD advice [28] but such information is fragmented and there is no central advice by the UK-NHS [29]. By contrast, in the USA, where HCD can be particularly common, community initiatives such as the Michigan Swimmer’s Itch Partnership (MISIP) have been developed to help mitigate local risk [30] and recently expanded to attempt to capture reports of HCD in the UK too (see https://swimersitch.info/map-your-itch-2023). Alternatively, in Denmark, an epidemiological baseline with an online national reporting system has been instigated [31].

## Conclusions

Despite surprisingly sparse literature, we have identified a clear need for better epidemiological surveillance, with near real-time reporting of HCD, in the UK. To do so, we suggest development of a national database overseen, for example, by the UKHSA or suitably engaged volunteer groups.

## Data Availability

All data are included as tables and figures within the article.

## Abbreviations

HCD: Human cercarial dermatitis
UK: United Kingdom
UKHSA: United Kingdom Health Security Agency
